# Dynamic changes in direction and frequency range of inter-areal cortical interactions revealed by non-parametric Granger causality

**DOI:** 10.1186/1471-2202-14-S1-P388

**Published:** 2013-07-08

**Authors:** Paul Tiesinga

**Affiliations:** 1 Department of Neuroinformatics, Donders Centre for Neuroscience, Radboud University Nijmegen, 6525 AJ Nijmegen, The Netherlands

## 

Models suggest that communication between brain areas could be mediated by oscillations in specific frequency bands. This hypothesis is supported by electrophysiological measurements wherein the direction and frequency of the interaction between brain areas varied during the different stages of a cognitive task. Frequency-resolved granger causality (GC) is used often for this analysis, but it has been derived for stationary linear processes, which may not be appropriate for brain activity. Our goal was to determine whether for nonlinear processes conditional GC could still distinguish direct connections from indirect ones and to what extent dynamic changes in the frequency & direction of interaction could be accurately detected. For this the properties of the GC determined parametrically, through fitting an autoregressive process of a fixed order, were compared to those obtained via non-parametric GC, which only uses the measured spectral matrix.

Oscillations in each cortical area were represented by a low-dimensional nonlinear model driven by white noise, whose standard deviation represented the level of activity in the respective areas. In addition, we analyzed the responses from two coupled networks of spiking neurons in the same way. We found that the direction of interaction between reciprocally connected cortical areas was determined by the level of activity, going from areas with high activity to those with low activity, which could be modulated on fast time scales. The frequency band of interaction was determined by the sending area.

For linear processes, the parametric GC had the lowest bias and variance compared to non-parametric GC, but was not appropriate for the nonlinear model and network model, because the oscillations could not be modeled accurately by autoregressive processes of a reasonable order. The non-parametric GC correctly represented the ground truth connectivity when multi-taper spectral estimates were used, but when multi-tapering made the spectral peaks too broad, artifacts emerged.

Taken together, the GC analysis revealed a simple rule for the direction and frequency band of communication between cortical areas, specifically "the loudest area gets heard", which can account for recent experimental results in which the frequency band of communication was direction dependent.

